# Supplementary material for: Prioritizing Solutions and Improving Resources among Young Pediatric Brain Tumor Survivors: Results of an Online Survey
Source: Curr Oncol. 2023 Sep 19;30(9):8586–601. doi: 10.3390/curroncol30090623 (PMC10527929; doi:10.3390/curroncol30090623)
Supplement: Supplementary file 1 [file curroncol-30-00623-s001.zip › Supplementary materials/Table S1- Long-form questionnaire – Young people version.pdf]

Table S1: Long-form questionnaire – Young people version

AJA-TC 2 Projet - Youth Version

Welcome to the online questionnaire for young survivors of childhood brain tumors ! N.B.:

The questionnaire lasts 30-45 minutes. You can edit your answers and return anytime to the survey to pick up where you left off. To do this, you must use the same electronic device that you started with until the end of the survey.

If you have some reading difficulties, you can either use screen readers equipped with a text-to- speech system available on your electronic devices or on search browsers, or a writing and reading assistive software (e.g. Word Q).

Let's start!

Who is this questionnaire for?

Young people living in Quebec aged 15 to 39 who have been diagnosed with a pediatric brain tumor and who have not received active treatments for at least one year (surgery, chemotherapy, radiotherapy).

* Are you a brain tumor survivor that meets the criteria mentioned above?

- Yes
- No

Thank you for your interest in participating. In this research we are only looking for young people aged 15 to 39 who have received a pediatric brain tumor diagnosis. However, if you have any comments or if would like to talk about this research, you can contact us (514-345-4931, ext. 3572). You can also share the link with the research information to those who might be interested and meet the criteria described in the first paragraph (in the section called "Who is this questionnaire for").

* How did you find out about this study?

- I received an information letter about the study via email
- I saw it on the website of an association or organization that helps people with cancer
- Someone told me about it
- Other (Specify)

Specity (parent, friends, healthcare professional, etc.)

____

____

Consent Form

APPROUVÉ PAR LE COMITÉ D'ÉTHIQUE

08 déc. 2020

#2021-2712

CHU SAINTE-JUSTINE

RESEARCH INFORMATION AND CONSENT FORM

Title: Overview of the design and development of post-treatment intervention protocols for adolescent and young adult brain tumor survivors

Principal investigator: Marco Bonanno, Psychologist, CHU Sainte-Justine

People responsible for the project at CHU Sainte-Justine:

- Serge Sultan, Full Professor, Université de Montreal, Director of the Psycho-Oncology Center, CHU Sainte-Justine
- Claude Julie Bourque, Scientific Coordinator, Clinical Ethics and Family Partnership Unit, CHU Sainte-Justine

Source of funding: Fonds d'intégration en psycho-oncologie (FIPO)

WHY ARE YOU INVITED TO PARTICIPATE IN THIS RESEARCH PROJECT?

The CHU Sainte-Justine research centre is developing research projects to improve the quality of life and access to care of children and adolescents living with and beyond cancer. In this context, we are taking the liberty of soliciting your participation. We invite you to read this information form and decide if you are interested in participating in this research project. It is important that you fully understand this form. Do not hesitate to ask questions. Take all the time you need to decide.

WHY ARE WE CONDUCTING THIS RESEARCH PROJECT?

Adolescents and young adults aged 15 to 39 who are brain tumor survivors often have specific needs in the health care settings. Because of cancer and its treatments, these young people are at higher risk than other populations of experiencing physical problems and psychological and social difficulties (e.g., success in studies and job search, social integration, anxiety, independence and self- esteem).

This project is the second phase of a research project that aims to put in place actionable plans to meet the needs of young survivors of brain cancer. In the first phase, we gathered and put together the perspectives of young survivors, parents and caregivers in order to generate ideas for possible solutions. In the current research phase, we are asking you to help us select the interventions that you consider to be a priority.

HOW MANY PEOPLE WILL PARTICIPATE IN THE RESEARCH PROJECT?

Approximately 60 participants will take part in this research project. They will be young people aged

between 15 and 39 years who have survived a brain tumor and parents who have a child between the ages of 15 and 39 years who has survived a brain tumor.

HOW WILL PARTICIPATION IN THIS RESEARCH PROJECT TAKE PLACE AND HOW LONG WILL PARTICIPATION TAKE?

The participants in this study are young survivors and parents of young survivors followed at the long-term follow-up clinic of the CHU Sainte-Justine or members of the Leucan association or members of the Canadian Brain Tumor Foundation.

You will be invited to complete an online questionnaire of approximately 30 minutes consisting of multiple choice and open-ended questions. At the start of the questionnaire, we will ask you to answer a few questions about your current situation and your cancer history. Subsequently, we will invite you to give your opinion on various interventions suggested to better meet the post-treatment needs of survivors along three themes: 1) interventions to promote the transition to resuming daily activities after treatments, 2) support and accompaniment interventions and, 3) information needs on the medical condition and on existing resources.

WHAT ARE THE RISKS?

There are no risks or inconveniences involved in participating in the study, except for the time required to complete the online questionnaire. When answering the questionnaire, remembering your experience could make you relive a difficult situation or make you feel like you need to talk about certain topics again. If this is the case, you can contact the principal investigator, whose contact details are below, to inform him of your needs. We can also provide you with the name of a professional who can provide you further support, if you wish.

ARE THERE ANY BENEFITS OF PARTICIPATING IN THIS RESEARCH PROJECT?

We hope that you will personally benefit from this research project, but we are not sure. A potential advantage that we are aiming for is that our work will lead to implementation of some of the proposals and recommendations based on your responses to the questionnaire. To meet this goal, based on your answers, we will set up a committee of people from different backgrounds (e.g., health professionals, stakeholders from the education and employment sectors, youth, parents and community organizations). The purpose of this committee will be to work on the necessary steps to make the interventions, which you have prioritized, usable and easy to access. We hope that the knowledge gained from this research project will be useful to other young people with cancer.

WHAT ARE THE OTHER FINANCIAL ASPECTS?

By participating in the study, you will be able to enter a raffle for a smart tablet valued at $ 200.

HOW IS CONFIDENTIALITY ENSURED?

As with any research project, unless you provide permission or as required by law, your answers are confidential. During your participation in this research project, the researcher in charge and his staff will collect and record your information in a research file. Only the information necessary for the proper conduct of the research project will be collected. This may include the following information: sex, age, type of cancer, treatments.

Your first and last names will not appear on the questionnaires. To ensure this, each research participant will be assigned a code and only the principal researcher or the person appointed for this purpose will have the list of participants and codes that have been assigned. This information will be kept in a locked cabinet located in a locked office (room B-469) at the CHU Sainte-Justine Research Center and will be under the responsibility of Dr. Serge Sultan.

The principal investigator of the study and his collaborators will use the data for research purposes in order to meet the scientific objectives of the research project described in this information and consent form. Data from the research project may be published in scientific journals or shared with others in scientific discussions. No publications or scientific communications will contain personally identifiable information. Otherwise, your permission will be requested beforehand. Research data will be kept for at least 10 years after the end of the project.

In order to verify that the research project is progressing well and to ensure your protection, the CHU Sainte-Justine Research Ethics Committee or a person appointed by the committee could consult your research file.

ARE YOU FREE TO PARTICIPATE?

Yes. Participation in this research project is voluntary. You are free to refuse to participate in this research project. You can withdraw from the research project at any time. If you do not participate in the research project or if you withdraw, this will not affect the quality of care offered to you or that could be offered to you in the future.

RESOURCE PERSON

If you have any questions regarding the research project or if you experience a problem that you believe relates to your participation in the project, you can contact the principal investigator for the project Marco Bonanno (514) 345-4931,

ext. 3572, or by email: marco.bonanno.hsj@ssss.gouv.qc.ca. For any information on your rights, you can contact the local service quality and complaints commissioner: CHU Sainte-Justine : 514-345-4749

WHERE CAN I OBTAIN MORE INFORMATION?

You can request a summary of the results of the research project; these will only be available when the project is completed. To this end, you can contact Marco Bonanno, Psychologist at the CHU Sainte- Justine and principal investigator of the project, at (514) 345-4931, ext. 3572, email: marco.bonannno.hsj@ssss.gouv.qc.ca. You can also contact Émélie Rondeau, Research Assistant at the Quality of Life and Psychosocial Oncology Laboratory, tel: (514) 345-4931, ext. 5123, email : emelie.rondeau.hsj@ssss.gouv.qc.ca

You can ask questions to the members of the research team mentioned above at any time.

RESEARCH ETHICS COMMITTEE

The CHU Sainte-Justine research ethics committee has approved this research project and is monitoring this project.

CONSEN T

Title of the research project: Overview of the design and development of post-treatment intervention protocols for adolescent and young adult brain tumor survivors

I understand the nature and process of the research project and I took note of the consent form. I had the opportunity to ask questions and receive the answers. Upon reflection, I agree to participate in this research project.

By signing this consent form, you do not waive any of your statutory rights. In addition, you do not release the investigators from their legal and professional responsibility in the event of a situation that would cause you harm.

By answering the following questionnaire, you agree to participate in this research project

Explanation of the Research

Young people who have had a brain tumor may experience difficulties after the treatments end. We would like to find ways to better help them.

In this questionnaire, you will find ideas to facilitate the return of these young people to activities of daily living after their treatments. They were offered by young people who have had a brain tumor, parents and hospital professionals. We would like to have your opinion on these ideas.

If needed, you can ask for help taking the quiz. For example, you can ask for help from someone you know (parents, friends, etc.). If you wish, you can also contact us to have someone help you complete the questionnaire, or to share your comments with us (514-345-4932, ext. 3572).

Thank you in advance for your help!

Sociodemographic Questions

The questionnaire is divided into two parts. In this first part, we are asking you to answer questions about your health, studies and work.

Health History

* In what year were you born?

* What is your gender?

- Male
- Female
- Other

* What is your cancer diagnosis?

- Astrocytoma (glioblastoma, optic pathway glioma, pilocytic astrocytoma)
- Medulloblastoma
- Ependymoma
- Germ cell tumor
- Craniopharyngioma
- I do not know
- Other (Specify)

In what year were you diagnosed with a brain tumor? (If you are not sure of the exact year of diagnosis, you can select the year that is closest)

What type(s) of treatment(s) did you receive? You can select all the choices that apply to your situation

- Radiotherapy
- Chemotherapy
- Surgery
- I do not remember

In what year did your treatments end? (If you are not sure of the exact year of diagnosis, you can select the one that is closest)

* In what type of health facility are you currently being followed for your cancer?

- Pediatric hospital
- Adult hospital
- I do not have any follow-up at a hospital, but I am followed by my family doctor
- Currently, I no longer have any follow-up for my cancer

* How often do you go to the hospital for your oncology follow-up?

- 6 months or less between each visit
- Over 6 months between each visit

* How often do you meet with your family doctor for your oncology follow-up?

- 6 months or less between each visit
- Over 6 months between each visit

As far as you know, in what year did you have your last follow-up in oncology?

If you compare yourself to others your age who haven't had cancer, how is your physical health?

Much worse	Worse	About the same	Better	Much better
◯	◯	◯	◯	◯

Do you have physical, psychological, cognitive or social difficulties as a result of your cancer and the treatments you have received?

- ☐ Yes
- ☐ No

What is the impact of these difficulties on your daily life? (For each type of difficulty described on the left, choose the answer that best reflects your opinion)

	Not at all important	Not really important	More or less important	Rather important	Very important
**Physical difficulties** (growth delay, difficulty hearing or seeing, weight problems, diabetes, etc.)	◯	◯	◯	◯	◯
**Psychological difficulties** (stress, depression, difficulty sleeping, difficulty in managing emotions, etc.)	◯	◯	◯	◯	◯
**Cognitive difficulties** (difficulty concentrating, memory difficulty, learning difficulty, etc.)	◯	◯	◯	◯	◯
**Social difficulties** (difficulty making friends, feeling isolated, difficulty being in a relationship, etc.)	☐	☐	☐	☐	☐

Studies

* While receiving treatment at the hospital for your cancer, did you have to stop going to school?

- Yes, I had to stop completely
- Yes, but there was a teacher or someone helping me with my studies at home
- No

For how long did you have to stop going to school?

* How would you describe your experience of returning to school after the end of your treatments (example: concentration, fatigue, material provided by the school, adapted lessons)?

Very easy	Easy	Slightly difficult	Very difficult	Does not apply to me
○	○	○	○	○

What helped you the most in your return ta school?

What was the hardest part of going back ta school for you?

AJA-TC 2 Projet - Youth Version

For how long did you have to stop going to school?

* How would you describe your experience of returning to school after the end of your treatments (example: concentration, fatigue, material provided by the school, adapted lessons)?

Very easy	Easy	Slightly difficult	Very difficult	Does not apply to me
○	○	○	○	○

What helped you the most in your return ta school?

What was the hardest part of going back ta school for you?

* How would you describe your experience of continuing your studies after the end of your treatments (e.g., concentration, fatigue, material provided by the school, adapted courses)?

Very easy	Easy	Slightly difficult	Very difficult	Does not apply to me
○	○	○	○	○

What helped you the most in the pursuit of your studies?

What was the most difficult for you in pursuing your studies?

AJA-TC 2 Projet - Youth Version

*Are you still studying at the moment?

- Yes
- No

AJA-TC 2 Projet - Youth Version

* At what level are you studying?

- Elementary
- High school or diploma of vocational studies (DEP)
- CÉGEP
- University
- Other (Specify)

AJA-TC 2 Projet - Youth Version

What type of program are you enrolled in?

- Diploma of vocational studies (DEP) – Regular
- Diploma of vocational studies (DEP) – Adult education
- High school diploma (DES) – Regular
- High school diploma (DES) – Adult education
- Certificate of professional studies (AEP) – Regular
- Certificate of professional studies (AEP) – Adult education
- Certificate of professional specialization (ASP)

* How many years have you been studying in this program full time or part time?

* What year of the program are you currently finishing?

- first year
- second year
- third year
- fourth year
- fifth year
- sixth year

AJA-TC 2 Projet - Youth Version

What type of program are you enrolled in?

- Quebec Diploma of College Studies (DEC) – Preuniversity studies
- Quebec Diploma of College Studies (DEC) – Technical studies
- Attestation of Collegial Studies (AEC)

* How many years have you been studying in this program full time or part time?

* What year of the program are you currently finishing?

- first year
- second year
- third year

What type of program are you enrolled in?

- Certificate
- Bachelor's/Undergraduate
- Masters
- Doctorate
- Other (Specify)

* How many years have you been studying in this program full time or part time?

* What year of the program are you currently finishing?

- first year
- second year
- third year
- fourth year
- fifth year

* How many years have you been studying in this program full time or part time?

* What year of the program are you currently finishing?

* What is the highest level of education that you have completed?

- Elementary
- High school or diploma of vocational studies (DEP)
- CÉGEP
- University
- Other (Specify)

AJA-TC 2 Projet - Youth Version

What is the last type of program you were enrolled in?

- Diploma of vocational studies (DEP) – Regular
- Diploma of vocational studies (DEP) – Adult education
- High school diploma (DES) – Regular
- High school diploma (DES) – Adult education
- Certificate of professional studies (AEP) – Regular
- Certificate of professional studies (AEP) – Adult education
- Certificate of professional specialization (ASP)

* How many years in total have you studied in this program full time or part time?

* What year of the program are you currently finishing?

- first year
- second year
- third year
- forth year
- fifth year
- sixth year

* Did you graduate?

- Yes
- No

AJA-TC 2 Projet - Youth Version

What is the last type of program you were enrolled in?

- Quebec Diploma of College Studies (DEC) – Preuniversity studies
- Quebec Diploma of College Studies (DEC) – Technical studies
- Attestation of Collegial Studies (AEC)

* How many years in total have you studied in this program full time or part time?

* What year of the program did you complete?

- first year
- second year
- third year

* Did you graduate?

- Yes
- No

What is the last type of program you were enrolled in?

- Certificate
- Bachelor's/Undergraduate
- Master
- Doctorate
- Other (Specify)

* How many years did you study in this program full-time or part-time?

* What year of the program did you complete?

- first year
- second year
- third year
- fourth year
- fifth year

* Did you graduate?

- Yes
- No

* How many years in total have you studied in this program full time or part time?

* What year of the program are you currently completing?

* Did you graduate?

- Yes
- No

Work

*Do you have a paid job right now?

- Yes
- No but I have worked in the past
- No I have never worked

* What type of work are you doing at the moment? You can select all the choices that apply to your current situation

- [ ] Employment with salary
- [ ] Internship with salary
- [ ] Internship without salary
- [ ] Volunteering

What is your job title? (example: daycare technician, store clerk, math instructor, mechanic)

How long have you worked in your current job?

- Less than one year
- Between one and five years
- More than five years

How many hours a week do you work?

- Less than 7 hours per week
- 7 to 30 hours per week
- Over 30 hours per week

* How would you describe the steps that allowed you to get your current job (example: write your CV, get interviews, answer questions during interviews)?

Very easy	Easy	Slightly difficult	Very difficult	I do not remember
○	○	○	○	○

What helped you the most in your efforts ta find a job?

What was the most difficult for you in your efforts ta find a job?

* How would you describe your ability to carry out your tasks at work (example: ability to concentrate, fatigue management, time required to complete a task)?

Very easy	Easy	Slightly difficult	Very difficult	I do not know
○	○	○	○	○

What has helped you the most ta succeed in your tasks at work?

What is the most difficult for you in carrying out your tasks at work?

* What was the last type of job you had?

- [ ] Employment with salary
- [ ] Internship with salary
- [ ] Internship without salary
- [ ] Volunteering

What was your job title? (example: daycare technician, store clerk, math instructor, mechanic)

For how long did you have this job?

- Less than one year
- Between one and five years
- More than five years

How many hours per week did you work on average?

- Less than 7 hours per week
- 7 to 30 hours per week
- Over 30 hours per week

* How would you describe the hiring process that led to your last job (example: writing your CV, getting interviews, answering questions during interviews)?

Very easy	Easy	Slightly difficult	Very difficult	I do not remember
○	○	○	○	○

What helped you the most in your efforts ta find a job?

What was the most difficult for you in your efforts ta find a job?

* How would you describe your ability to carry out tasks in your last job (example: ability to concentrate, fatigue management, time required to complete a task)?

Very easy	Easy	Slightly difficult	Very difficult	I do not remember
○	○	○	○	○

What helped you the most ta succeed in your tasks at work?

What was the most difficult for you in carrying out your tasks at work?

Which sentence best represents your employment situation at the moment?

- I am looking for a job (for example I send CVs, I do interviews)
- I am not looking for a job
- I stopped looking for a job because it was difficult to find another job
- I don't feel able to work at the moment
- Other (Specify)

AJA-TC 2 Projet - Youth Version

* Choose the sentences that correspond to your current situation.

- ☐ I consider that I am still too young to work
- ☐ I receive financial support from the government (e.g., social assistance or unemployment insurance)
- ☐ My health state does not allow me to work
- ☐ I tried to find a job, but it was difficult to get one
- ☐ Other (Specify)

What was difficult in your efforts ta find a job?

Family situation

* What is your current family situation? You can select all the choices that apply to your situation

- [ ] Single
- [ ] In a relationship
- [ ] Separated/divorced
- [ ] With child(ren)
- [ ] Waiting for a child
- [ ] Other (Specify)

* What is your main place of residence?

- ( ) I live with my parents or with one of my parents
- ( ) I live alone
- ( ) I live with roommates (example: friends, brother, sister)
- ( ) I live with my partner

* What is your current main source of income? (choose only the most important source of income)

- ( ) My job
- ( ) A government program (social assistance, unemployment, other)
- ( ) Support from my parents or family
- ( ) Support from my spouse
- ( ) Loans and scholarships for studies
- ( ) No income

Solutions

This is to remind you that on the following pages you will find ideas that were provided to us by young people who have had a brain tumor to facilitate the return to everyday activities after the treatments.

Based on your experience, we want to know if you find these solutions to be useful and important for young people who have had a brain tumor. While you are answering the questionnaire, if you have any other ideas or comments that come to mind, please share them with us in the spaces provided.

Return to everyday activities

Cancer and some treatments for brain tumors (such as chemotherapy, radiation therapy, or surgery) can cause difficulties with everyday activities. Youth, parents, and professionals have told us that sometimes survivors have trouble making new friends, studying or working after their cancer.

Here are some ideas they suggested. We would like to know how helpful you find these suggestions.

For each proposed solution, select the answer that best represents your opinion.

Solution 1 : Once the treatments are finished, be evaluated by hospital professionals (example: neuropsychologist, occupational therapist, physiotherapist) to better understand your needs for the return to your daily activities (example: school, work, social relations).

According to you, this solution would be

not at all helpful	slightly helpful	pretty helpful	very helpful	I do not know
○	○	○	○	○

Free comment

Solution 2 : Receive advise and recommendations from hospital professionals to help you explain your situation and request appropriate services at school or at work.

According to you, this solution would be

not at all helpful	slightly helpful	pretty helpful	very helpful	I do not know
○	○	○	○	○

Free comment

Solution 3 : Have a hospital professional contact the school or workplace and offer recommendations for services tailored to your needs (example: having access to a computer in class, more time for homework or work tasks, place with less noise to better concentrate)

According to you, this solution would be

not at all helpful	slightly helpful	pretty helpful	very helpful	I do not know
○	○	○	○	○

Free comment

Solution 4 : Participate in a meeting (intervention plan or end of treatment plan) with members of your family, hospital professionals and workers from your school or work, to discuss your needs and organize the necessary services for your return to school or work.

According to you, this solution would be

not at all helpful	slightly helpful	pretty helpful	very helpful	I do not know
○	○	○	○	○

Free comment

Solution 5 : After resuming your daily activities, have access to regular follow-up by hospital professionals if needed (example: occupational therapist, physiotherapist, psychologist, neuropsychologist, social worker) to receive support or evaluations.

According to you, this solution would be

not at all helpful	slightly helpful	pretty helpful	very helpful	I do not know
○	○	○	○	○

Free comment

Support

The young people we met told us that sometimes they feel alone or misunderstood. Once the treatments were over, they would have needed support to better understand their condition and better communicate with their old friends and make new friends.

Here are some ideas they suggested on that topic. We would like to know how helpful you find these suggestions.

For each proposed solution, select the answer that best represents your opinion.

Solution 6 : Meet other young people who are going through a similar situation to talk. This could be done over the internet on a secure site or during an activity, for example going to a restaurant.

According to you, this solution would be

not at all helpful	slightly helpful	pretty helpful	very helpful	I do not know
○	○	○	○	○

Free comment

Solution 7 : Meet with a hospital professional who can answer your questions about friendships, romantic relationships, or sexuality.

According to you, this solution would be

not at all helpful	slightly helpful	pretty helpful	very helpful	I do not know
○	○	○	○	○

Free comment

Solution 8 : Being able to count on the support of a professional from the hospital or another former patient to help you cope with the difficulties of daily life following your cancer (example: explaining your difficulties to others, giving yourself ideas on how to make new friends).

According to you, this solution would be

not at all helpful	slightly helpful	pretty helpful	very helpful	I do not know
○	○	○	○	○

Free comment

Solution 9 : Receive advice from a professional who helps you choose an area of study or employment, taking into account your strengths, limitations and interests, and who gives you advice at different stages of your studies/employment.

According to you, this solution would be

not at all helpful	slightly helpful	pretty helpful	very helpful	I do not know
○	○	○	○	○

Free comment

Solution 10 : Participate in school or professional orientation group meetings to discuss studies and work with other young people who are living a situation similar to yours.

According to you, this solution would be

not at all helpful	slightly helpful	pretty helpful	very helpful	I do not know
○	○	○	○	○

Free comment

Information

According to the young people we met, after the end of treatments it is important to receive adequate information when you experience a need. It can be all kinds of information, such as precise details on the long-term effects of treatments or advice on how to better adapt to everyday activities.

Here are some ideas they suggested on that topic. We would like to know how helpful you find these suggestions.

For each proposed solution, select the answer that best represents your opinion.

Solution 11 : Have a list of places and services where you can find suitable equipment for everyday life to meet your difficulties (example: an adapted car, stores for adapted shoes or adapted clothing)

According to you, this solution would be

not at all helpful	slightly helpful	pretty helpful	very helpful	I do not know
○	○	○	○	○

Free comment

Solution 12 : Have documents that describe any difficulties that you might encounter after finishing your treatments and the best solutions and resources available to help you overcome them.

According to you, this solution would be

not at all helpful	slightly helpful	pretty helpful	very helpful	I do not know
○	○	○	○	○

Free comment

Solution 13 : Have information on resources and solutions available to help you in school (example: having a computer, having more time during exams).

According to you, this solution would be

not at all helpful	slightly helpful	pretty helpful	very helpful	I do not know
○	○	○	○	○

Free comment

Solution 14 : Have information on the resources and solutions available to help you at work (example: adapted chair, flexible schedule).

According to you, this solution would be

not at all helpful	slightly helpful	pretty helpful	very helpful	I do not know
○	○	○	○	○

Free comment

General questions on the questionnaire

If you have other solutions or ideas to help young people who are going through a situation similar to yours, you can write them here in your own words:

Did you get help to answer the questionnaire?

- Yes
- No

Can you tell us the relationship to the person who helped you (example: your brother, a friend, your mother)?

Further research, other projects and participation in a raffle

* Would you agree to participate in another phase of this study over the next two years? We could, for example, invite you to answer other questionnaires or attend meetings.

- [ ] **Yes**: If we communicate with you, you will always be free to accept or refuse
- [ ] **I don't know:** I will decide when you contact me
- [ ] **No**

* We may set up projects in which we would like to have young survivors as collaborators and advisers. If we establish such projects, would you like us to invite you to participate in this kind of activity?

- [ ] **Yes:** If we communicate with you you will always be free to accept or refuse
- [ ] **I don't know**: I will decide when you contact me
- [ ] **No**

* Participation in this study gives you the right to participate in a raffle for a smart tablet. Would you like to register?

- [ ] **Yes**
- [ ] **No**

If you answered "**Yes**" or "**I don't know**" to one of the last three questions, we need your contact information to be able to contact you.

Last name

First name

Address

Address 2

City

Province

Zip code

Country

Email address

Phone number

Thank you for your participation!

If you need more information about the study you just participated in, here are the contact details of the person you can reach to ask your questions:

Marco Bonanno
CHU Sainte-Justine
Email : marco.bonanno.hsj@ssss.gouv.qc.ca
Phone number : (514) 345-4931 (3572)
