# Supplementary material for: Prioritizing Solutions and Improving Resources among Young Pediatric Brain Tumor Survivors: Results of an Online Survey
Source: Curr Oncol. 2023 Sep 19;30(9):8586–601. doi: 10.3390/curroncol30090623 (PMC10527929; doi:10.3390/curroncol30090623)
Supplement: Supplementary file 1 [file curroncol-30-00623-s001.zip › Supplementary materials/Table S2- Experiences and difficulties of survivors in aftercare in health, school and work’s domain.pdf]

Table S2: Experiences and difficulties of survivors in aftercare in health, school and work's domain.

Domains	Topics	Selection	Young survivors N (%)	Parents N (%)	Total N (%)
**Health**	Perceived health status compared with peers	Much worse	11 (25.6)	9 (37.5)	20 (29.9)
		Worse	19 (44.2)	9 (37.5)	28 (41.8)
		About the same	12 (27.9)	6 (25)	18 (26.9)
		Better	1 (25.6)	-	1 (1.5)
	Difficulties cancer related	No	8 (19)	2 (8)	10 (14.9)
		Yes	34 (81)	23 (92)	57 (85.1)
**School**	Current Study attendance	No	20 (47.6)	14 (58.3)	34 (51.5)
		Yes	22 (52.4)	10 (41.7)	32 (48.5)
	Past study level	Elementary	1 (4.5)	-	1 (3.1)
		Hight school or DEP	6 (27.3)	3 (30)	9 (28.1)
		CEGEP*	6 (27.3)	4 (40)	10 (31.3)
		University	4 (18.2)	-	4 (12.5)
		Other	5 (22.7)	3 (30)	8 (25)
	Current study level	Elementary	-	-	-
		Hight school or DEP	5 (25)	11 (78.6)	16 (47.1)
		CEGEP*	3 (15)	1 (7.1)	14 (11.8)
		University	8 (40)	2 (14.3)	10 (29.4)
		Other	4 (20)	-	4 (11.2)
	School break during treatments	No	10 (23.3)	6 (25)	16 (23.9)
		Yes, completely	22 (51.2)	7 (29.2)	29 (43.3)
		Yes, with a teacher at home	11 (25.6)	11 (45.8)	22 (32.8)
	School attendance (without break during treatments)	Very Easy	-	-	-
		Easy	2 (22.2)	2 (33.3)	4 (26.7)
		Slightly difficult	5 (55.6)	1 (16.7)	6 (40)
		Very difficult	2 (22.2)	3 (50)	5 (33.3)
	Back to school (with teacher at home during treatments)	Very Easy	-	-	-
		Easy	-	-	-
		Slightly difficult	7 (63.6)	6 (54.5)	13 (59.1)
		Very difficult	4 (36.4)	5 (45.4)	9 (40.9)
	Back to school (after full break during treatments)	Very Easy	1 (4.8)	-	1 (3.6)
		Easy	1 (4.8)	-	1 (3.6)
		Slightly difficult	5 (23.8)	-	5 (17.8)
		Very difficult	14 (66.7)	7 (100)	21 (75)
**Work**	Current work situation	Never worked	5 (11.9)	6 (25)	11(16.7)
		Already worked	15 (35.7)	8 (33.3)	23 (34.8)
		Presently working	22 (52.4)	10 (41.7)	32 (48.5)
	Years of work	Less than one year	16 (45.7)	6 (33.3)	22 (41.5)
		Between one and five years	15 (42.8)	11 (61.1)	26 (49)
		More than five years	4 (11.5)	1 (5.6)	5 (9.5)
	Work hours	Less than 7 h/week	2 (5.6)	6 (33.3)	8 (14.8)
		7 to 30h/week	23 (63.8)	8 (44.5)	31 (57.4)
		Over 30h	11 (30.6)	4 (22.2)	15 (27.8)
	Experience in finding a job	Very Easy	5 (14.3)	3 (17.6)	8 (16)
		Easy	9 (25.7)	5 (29,4)	14 (27.5)
		Slightly difficult	13 (37)	2 (11.8)	15 (24.4)
		Very difficult	8 (23)	7 (41.2)	15 (32.1)
		Very easy	7 (19.4)	2 (11.1)	9 (15.3)

Ability to carry out tasks	Easy	11 (30.6)	5 (27.8)	16 (29.2)
	Slightly difficult	13 (36)	7 (38.9)	20 (37.4)
	Very difficult	5 (14)	4 (22.2)	9 (18.1)

*CÉGEP is a degree of general and professional study in Quebec education system following High school that precede and can prepare to the University
