# Supplementary material for: Prioritizing Solutions and Improving Resources among Young Pediatric Brain Tumor Survivors: Results of an Online Survey
Source: Curr Oncol. 2023 Sep 19;30(9):8586–601. doi: 10.3390/curroncol30090623 (PMC10527929; doi:10.3390/curroncol30090623)
Supplement: Supplementary file 1 [file curroncol-30-00623-s001.zip › Supplementary materials/Table S3 - Bonferroni post hoc test.pdf]

Table S3: Bonferroni post hoc test

P-values following repeated measure ANOVA comparing means of endorsing 14 solutions on a 0 (not at all helpful) to 3 (very helpful) scale (n = 63)

Solutions	1	2	3	4	5	6	7	8	9	10	11	12	13	14
1														
2	1.000													
3	1.000	1.000												
4	0.887	0.524	1.000											
5	1.000	1.000	1.000	0.056										
6	1.000	1.000	1.000	1.000	0.983									
7	0.002	0.003	0.006	1.000	< 0.001	0.162								
8	0.722	1.000	1.000	1.000	0.307	1.000	0.884							
9	1.000	1.000	1.000	1.000	1.000	1.000	0.063	1.000						
10	0.074	0.110	0.145	1.000	0.007	1.000	1.000	1.000	1.000					
11	0.761	0.856	1.000	1.000	0.209	1.000	1.000	1.000	1.000	1.000				
12	1.000	1.000	1.000	1.000	1.000	1.000	0.019	1.000	1.000	0.900	1.000			
13	1.000	1.000	1.000	0.099	1.000	1.000	0.008	1.000	1.000	0.070	0.587	1.000		
14	1.000	1.000	1.000	1.000	1.000	1.000	0.477	1.000	1.000	1.000	1.000	1.000	1.000	

Note.: ANOVA with a Greenhouse-Geisser correction ($F(8.401) = 5.459$, $p < 0.001$); Post-hoc statistically significant differences (#7<#1; #7<#2; #7<#3; #7<#5; #7<12; #7<#13, and #10<#5)
